# Sociodemographic and COVID-Related Predictors for Mental Health Condition of Mainland Chinese in Canada Amidst the Pandemic

**DOI:** 10.3390/ijerph19010171

**Published:** 2021-12-24

**Authors:** Linke Yu, Mariah Lecompte, Weiguo Zhang, Peizhong Wang, Lixia Yang

**Affiliations:** 1 Department of Psychology, Ryerson University, Toronto, ON M5B 2K3, Canada; ylinke@ryerson.ca (L.Y.); mlecompte@ryerson.ca (M.L.); 2 Department of Sociology, University of Toronto Mississauga, Mississauga, ON L5L 1C6, Canada; weiguo.zhang@utoronto.ca; 3 Dalla Lana School of Public Health, University of Toronto, Toronto, ON M5T 3M7, Canada; pwang@mun.ca; 4 Division of Community Health and Humanities, Faculty of Medicine, Memorial University of Newfoundland, St. John’s, NL A1B 3V6, Canada

**Keywords:** COVID-19, mental health, Mainland Chinese, sociodemographic predictors, COVID-19-related predictors

## Abstract

The current study investigates the mental health condition of Mainland Chinese in Canada and identifies the associated sociodemographic and COVID-19-related predictors. A sample of 471 Mainland Chinese aged 18 or older completed an online survey that collected information on demographics, experience, cognition, and behaviours related to the COVID-19 pandemic and mental health condition. Mental health condition was assessed with the Depression, Anxiety, and Stress Scale-21 (DASS-21) for the depression, anxiety, and stress levels of Mainland Chinese during the pandemic. Moderate to severe depression, anxiety, and stress levels were respectively reported by 11.30%, 10.83%, and 5.10% of respondents. Univariate analysis of variance models (ANOVAs) were conducted to assess mental health condition variance as stratified by independent sociodemographic- or COVID-19-related explanatory variables, to identify possible predictors to be entered into the subsequent regression models. The regression models identified age, income level, health status, and perceived discrimination as significant sociodemographic predictors (absolute value of *β*s = 1.19–7.11, *p*s < 0.05), whereas self-infection worry, attitude towards Canadian measures, information confusion, food/goods stocking, and room cleaning/sanitizing were identified as significant COVID-19-reltaed predictors (absolute value of *β*s = 1.33–3.45, *p*s < 0.05) for mental health outcomes. The results shed light on our understanding of the major factors associated with the mental health condition of Mainland Chinese in Canada during the COVID-19 pandemic.

## 1. Introduction

The COVID-19 pandemic has widely spread across the world. Since March 2020, Canada’s federal government implemented strict public health measures, including lockdowns, social distancing measures, and closing most public facilities, to restrict the spread of the virus [1,2]. Along with these measures, it has been evidenced that the COVID-19 outbreak has a detrimental effect on the psychological well-being of Canadians [3,4]. In a global study, it was found that there was a significantly larger number of depression and stress symptoms reported in Canada than in other countries [5]. A survey of a nationally representative Canadian sample indicated that reported depression symptoms showed over a two-fold increase, and that anxiety symptoms showed nearly a four-fold increase [6] during the pandemic. Due to rising racial discrimination during the pandemic, and their close ties to China, Mainland Chinese in Canada may be particularly vulnerable to the mental health impacts of the COVID-19 pandemic. The current study sought to examine the mental health condition of Mainland Chinese in Canada and to identify the related sociodemographic and COVID-19-related predictors at the early stage of the pandemic in Canada (April–June 2020).

### 1.1. Psychological Outcomes of the COVID-19 Outbreak among Mainland Chinese

The COVID-19 pandemic has had a detrimental impact on psychological wellbeing. It has been suggested that the social isolation during the pandemic was strongly correlated with mental health symptoms [7]. Mainland Chinese represent a vulnerable population for mental health concerns, given their social/cultural stigma towards mental health, barriers to accessing related services, and their preference for traditional Chinese health practices [8,9,10]. These issues are likely to be exacerbated by the quarantine and social distancing practices. Moreover, the effect of COVID-19 has been found to be prolonged on the Chinese population. For example, Mainland Chinese reported higher post-traumatic stress symptoms than other nationalities (e.g., Spanish and American) during the COVID-19 pandemic [11,12,13]. It is therefore important to understand the mental health condition of Mainland Chinese during the pandemic, especially in such a multicultural society as Canada.

A study by Wang et al. [14], conducted in Mainland China during the initial outbreak of COVID-19, showed that 53.8% of respondents reported moderate-to-severe psychological distress. Among all respondents, 16.5% reported moderate-to-severe depression, 28.8% reported moderate-to-severe anxiety, and 8.1% reported moderate-to-severe stress [14]. These findings suggest that the COVID-19 outbreak has a negative impact on people in Mainland China. However, there is a paucity of research on the mental health condition of Mainland Chinese in Canada during the pandemic. Mainland Chinese in Canada may face racial discrimination when adapting to Western culture [15]. It has been reported that immigrants are at a higher risk for discrimination, which is related to their poor mental health outcomes [16]. Recent poll data showed that 64% of new citizens and 69% of Canadians of color were worried about an increased incidence of discrimination and prejudice due to COVID-19 [17]. Given the initial occurrence of COVID-19 in Mainland China, Chinese residents in Canada might be especially vulnerable to and impacted by the rising perceived and experienced discrimination. Based on the Canadian survey data in May 2020, harassment or attacks against Chinese Canadians based on race, ethnicity, and skin color increased by 30% since the start of the COVID-19 outbreak, and this was the highest among all racial groups [18]. As a result, the mental health of Chinese residents in Canada may be particularly jeopardized during the pandemic.

In light of these considerations, the primary goal of the current study was to fill in the gap in the literature to investigate the mental health condition of Mainland Chinese living in Canada during the COVID-19 pandemic. The secondary goal was to identify the individual (i.e., sociodemographic) and environmental (i.e., COVID-19-related) predictors for their mental health condition.

### 1.2. Sociodemographic and COVID-19-Related Predictors of Mental Health Condition

Some sociodemographic variables have been identified as predictors for psychological distress level during the COVID-19 pandemic [14,19]. Studies across the globe have identified some mental health risk factors, such as gender, age, education, health condition, and financial status [5,14,19,20,21,22,23,24]. Specifically, individuals who are females, at a younger age, and have a lower education level have been found to be more likely to experience greater psychological distress relative to their corresponding counterparts [5,14,19,20,21,22,23,24]. For example, a global survey across 26 countries identified the following mental health risk factors: being a woman, younger age, lower education level, being unmarried/without a partner, living with more children, and living in a country heavily impacted by the COVID-19 pandemic [25]. Similarly, another global study found that being a woman and of a young age (18–24 years) was related to a higher risk for experiencing depression, anxiety, and distress symptoms [5].

The psychological distress may be further exacerbated by the increased prejudice and discrimination [25,26]. Research with Polish and UK-based samples found a greater prejudice against those from nations that have been especially impacted by the virus, such as China and Italy [27]. Similarly, racism has been present in news coverage of COVID-19 [28], and this partially accounts for the rise in perceived racial discrimination and anti-Asian actions against Chinese [26]. It is predicted that perceived and experienced discrimination will jeopardize their mental health condition. In this context, it is urgent, timely, and important to understand how likely the growing concerns of discrimination and racism would predict the mental health condition of Mainland Chinese in Canada.

On the other hand, COVID-19-related experience, cognition, and behaviours may also be associated with the mental health status during the pandemic. It has been revealed that experiencing physical symptoms related to a COVID-19 infection was associated with self-reported experiences of traumatic distress, depression, anxiety, and stress symptoms [14]. Additionally, Traunmüller and colleagues [21] found that the proportionally overly worried individuals were more likely to report depression, anxiety, and distress symptoms. Similar results were also revealed in Wang and colleagues [14]. However, the results on the relationship between precautious behaviour endorsement and mental health are mixed and inconclusive. For example, Wang et al. [14] found that experience with COVID-19 symptoms was related to greater depression, anxiety, and stress, whereas engagement in specific behaviours (e.g., wearing a face mask, frequent hand washing) was related to lower psychological distress. Similarly, González Ramírez and colleagues [19] found higher levels of psychological distress in those who experienced a greater change in normal life routine and endorsed fewer precautious activities. In contrast, Traunmüller et al. [21] found higher levels of psychological distress in those who engaged in more precautionary behaviours. Similar results were found during the SARS outbreak in 2003 [29]. Given the inconsistencies, further research is needed to explore the relationship of COVID-19-related variables and the mental health condition of Mainland Chinese in Canada. This will provide insights on the related policies and measures to effectively combat the spread of the virus, and its mental health outcomes.

### 1.3. Present Study

The current study examined the mental health condition of Mainland Chinese in Canada during the COVID-19 pandemic and identified sociodemographic and COVID-19-related predictors. Specifically, this study aimed (1) to examine the mental health condition (i.e., depression, anxiety, and stress levels) of Mainland Chinese in Canada during the COVID-19 pandemic, and (2) to identify sociodemographic and COVID-19-related predictors for mental health outcomes in this population. To address these objectives, an online survey was designed and administered to a convenient sample of Mainland Chinese living in Canada, mainly in Ontario. Following Wang et al. [14], we used the Depression, Anxiety, and Stress Scale-21 (DASS-21) to assess mental health condition [30]. In light of the literature, we hypothesized that Mainland Chinese in Canada might be more vulnerable to depression, anxiety and stress during the pandemic, compared to the data collected in Mainland China [14]. It is also predicted that the mental health condition would be predicted by critical sociodemographic variables, such as age, gender, education, employment/financial/health status, perceived or experienced discrimination, and COVID-19-related worry, information perception, as well as precautionary behavioral measure endorsement.

## 2. Materials and Methods

### 2.1. Participants

A random sampling approach was used to recruit participants primarily through a variety of on-line platforms, such as WeChat (i.e., the most popular social media platform among Chinese across the globe), websites, and emails. A convenient sample of 1078 participants attempted to complete this online survey, but only 656 were eligible based on the inclusion criteria: (1) at least 18 years old and migrated from China; (2) have lived or plan to live in Canada for more than four weeks; (3) can read and write Chinese. The current report specifically focused on mental health condition assessed by the Depression, Anxiety, and Stress Scale-21 (DASS-21) [30]. There were 185 participants (28.2%) who failed to complete half of the items (i.e., 4 out of 7 items) in each subscale, and who were thus excluded from the final analysis, resulting in a final sample of 471 participants. As per convention, missing values (2.1% in depression, 1.9% in anxiety, and 1.7% in stress) were replaced by the average on each subscale for each participant. Please note that we also analyzed the data without the cases with missing values, and the results remained largely the same. For the sake of statistical power, we included them in the final analysis. The majority of participants in the final sample were female (70.49%), Ontario residents (80.04%), with a university degree or higher (74.31%), and with a middle or higher financial status (71.34%). Table 1 displays the sample characteristics.

**Table 1:** Sample characteristics and group differences in DASS-21 scores, *N* = 433.

Variables		*N* (%)	Depression			Anxiety			Stress		
			M (SD)	F	*p*	M (SD)	F	*p*	M (SD)	F	*p*
Age group	<35	44 (10)	4.63 (5.31)	4.10	**0.017**	3.09 (3.62)	2.91	**0.055**	5.14 (5.71)	4.35	**0.014**
	35–64	354 (82)	5.40 (7.01)			4.01 (5.94)			6.52 (7.37)		
	≥65	35 (8)	3.68 (5.91)			2.51 (3.61)			3.69 (5.25)		
Gender	Female	324 (75)	5.39 (6.99)	0.05	0.826	4.03 (5.65)	0.23	0.633	6.25 (7.22)	0.29	0.593
	Male	109 (25)	4.16 (5.83)			2.81 (5.02)			5.18 (6.58)		
Years in Canada	0–5 years	70 (16)	5.17 (5.46)	1.88	**0.153**	3.29 (4.36)	2.10	**0.124**	5.81 (5.83)	2.48	**0.085**
	6–15 years	147 (34)	5.73 (8.07)			4.26 (6.53)			6.65 (8.31)		
	>15 years	216 (50)	4.60 (5.99)			3.50 (5.07)			5.61 (6.44)		
Marital status	Married /Partnered	360 (83)	4.81 (6.49)	1.43	0.232	3.59 (5.37)	0.19	0.661	5.85 (6.88)	0.22	0.643
	Other	73 (17)	6.25 (7.55)			4.22 (5.91)			6.55 (7.57)		
Education	College and under	104 (24)	6.12 (8.01)	0.32	0.728	4.66 (6.90)	0.29	0.750	6.75 (8.03)	0.08	0.926
	University	170 (39)	5.40 (6.96)			3.76 (5.43)			6.03 (7.16)		
	Graduate	159 (37)	4.01 (5.19)			3.02 (4.41)			5.42 (6.17)		
Living arrangement	Alone	21 (5)	7.92 (6.83)	0.41	0.663	5.64 (5.19)	0.31	0.734	8.95 (7.38)	0.81	0.445
	2 people	89 (20)	4.88 (6.64)			3.31 (4.71)			5.70 (7.11)		
	3 or above	323 (75)	4.97 (6.71)			3.70 (5.67)			5.87 (6.95)		
The region of residence	Ontario	354 (82)	5.18 (6.75)	0.885	0.347	3.83 (5.72)	1.06	0.303	6.18 (7.07)	2.63	**0.106**
	Other provinces	79 (18)	4.74 (6.59)			3.23 (4.37)			5.18 (6.78)		
Employment status	Employed	207 (48)	4.72 (6.68)	0.784	0.457	3.66 (5.71)	0.79	0.453	6.24 (7.39)	0.73	0.481
	Self-employed	90 (21)	4.64 (5.19)			3.23 (4.30)			5.52 (5.53)		
	Others	136 (31)	5.79 (7.44)			4.02 (5.75)			5.90 (7.27)		
Income level	Low	113 (26)	6.52 (8.43)	2.23	**0.110**	4.60 (7.06)	1.40	0.247	6.67 (8.45)	0.55	0.58
	Middle	189 (44)	4.82 (6.04)			3.70 (4.70)			5.94 (6.38)		
	High	131 (30)	4.16 (5.63)			2.94 (4.95)			5.46 (6.60)		
Health status	Poor	144 (33)	10.10 (8.65)	126.83	**0.000**	7.54 (7.66)	102.01	**0.000**	11.11 (8.79)	123.98	**0.000**
	Good (>3)	289 (67)	2.79 (3.85)			1.96 (2.67)			3.63 (4.36)		
Religion	No	232 (54)	5.34 (6.79)	1.01	0.365	3.83 (5.53)	0.63	0.535	5.91 (7.02)	0.05	0.951
	Christianity /Catholicism	151 (35)	4.49 (6.10)			3.26 (5.18)			5.66 (6.46)		
	Others	50 (11)	5.81 (8.11)			4.60 (6.07)			7.32 (8.54)		
Perceived discrimination	Agree	236 (55)	6.39 (7.45)	6.82	**0.001**	4.62 (6.22)	5.15	**0.006**	7.42 (7.72)	7.97	**0.000**
	Neutral	140 (32)	4.14 (5.93)			3.14 (4.64)			5.00 (6.02)		
	Disagree	57 (13)	2.31 (3.68)			1.53 (2.85)			2.77 (4.51)		

Note. Bold *p* values (*p* ≤ 0.20) refer to the variables entered in the regression models in Table 2.

**Table 2:** Linear regression models for mental health outcomes (i.e., depression, anxiety and stress).

Predictors	Depression (*N* = 471)		Anxiety (*N* = 471)		Stress (*N* = 426)	
	β	95% CI	β	95% CI	β	95% CI
Age group						
<35 (reference)						
35–64	0.71	−0.81, 2.23	0.43	−0.84, 1.70	1.65 *	0.01, 3.29
≥65	−1.49	−3.84, 0.85	−1.05	−3.02, 0.92	−1.20	−3.73, 1.34
Years in Canada						
0–5 years (reference)						
6–15 years	0.62	−0.91, 2.15	0.97	−0.32, 2.26	0.28	−1.47, 2.02
>15 years	−0.59	−2.11, 0.94	−0.07	−1.33, 1.19	−0.97	−2.65, 0.71
The region of residence						
Ontario (reference)	X	X	X	X		
Other provinces	X	X	X	X	−0.49	−1.04, 0.06
Income level						
Low (reference)						
Middle	−1.76 **	−3.04, −0.49	X	X	X	X
High	−1.68 *	−3.13, −0.22	X	X	X	X
Health status						
Poor (reference)						
Good (>3)	−7.01 ***	−8.13, −5.88	−5.42 ***	−6.36, −4.48	−7.09 ***	−8.29, −5.88
Perceived discrimination						
Agree (reference)						
Neutral	−1.71 **	−2.86, −0.56	−1.09 *	−2.06, −0.12	−1.52 *	−2.76, −0.28
Disagree	−2.97 ***	−4.53, −1.41	−2.11 **	−3.42, −0.79	−3.12 ***	−4.79, −1.45

Note: Listwise method was used to handle missing values. CI = Confidence of Interval. “X” cells refer to the variables not included in the regression model based on the ANOVA analysis in Table 1. * *p* < 0.05, ** *p* < 0.01, *** *p* < 0.001.

### 2.2. The Survey

The survey was built in Qualtrics^TM^ (Qualtrics, Provo, UT, USA.) and delivered on-line in Mandarin during the peak period of the first wave of the COVID-19 pandemic in Canada, from April 25 to June 10, 2020. The survey collected information on participants’ perception, experience, and behavioral measures related to COVID-19, their mental health condition (e.g., DASS-21), and their sociodemographic information. The current report sought to describe the mental health profile of Mainland Chinese in Canada and identify its associated sociodemographic and COVID-19 perception/experience/behaviour predictors.

### 2.3. Explanatory Variables

For the purpose of our study, we selected two groups of explanatory variables. The first group included sociodemographic variables such as age, gender, years in Canada, marital status, education level, living arrangement (i.e., number of people living together), the region of residence, employment status, income level, health status (physical, mental, and sleep quality), religion, and perceived discrimination (“Do you think Chinese Canadians will experience prejudice and discrimination because of COVID-19?”). All the questions were multiple-choice/categorical and allowed text-entry if none of the choices applied (see Table 1). The second group included items on COVID-19-related experience, perception, and behaviours, including contact history, risk perception, perceived behaviour measure effectiveness, and precautionary measure endorsement (see Table 3). Contact history was assessed with the following questions: “Have you ever interacted with any confirmed/suspected COVID-19 cases?” (exposure); “Did you or any of your family members develop symptoms like fever and cough in the past two weeks?” (symptom); “Have any of your relatives/friends/colleagues been diagnosed with COVID-19?” (other-contraction). Risk perception was assessed with the following questions: “How likely would you be infected with the COVID-19?” (self-infection prediction), “do you worry that yourself being infected with COVID-19?” (self-infection worry), “do you worry that your family members being infected with COVID-19?” (family-infection worry), “What is your attitude towards the Canadian measures battling against the COVID-19?” (attitude towards Canadian measures), “Do you think that the COVID-19 pandemic is a real threat?” (threat perception), “Are you confused or doubtful about the authenticity of the COVID-19-related information you received?” (information confusion). Perceived behaviour measure effectiveness was assessed with such behaviour measures as mask wearing, taking Vitamin C or other supplements, and gargling with salt water. All the questions mentioned so far were based on a Likert scale. Finally, precautionary measure endorsement was assessed with the “YES/NO” responses to a list of measures, such as food/goods stocking and room cleaning/sanitizing.

### 2.4. Outcome Variables

Similar to Wang et al. [14], the dependent variables included depression, anxiety, and stress scores, as assessed with the DASS-21, which includes 21 symptom items based on a 4-point Likert scale, ranging from “0” (did not apply to me at all) to “3” (applied to me most of the time) in the past week, including today. The score was the double of the sum score of the 7 items for each of the three subscales: depression (items 3, 5, 10, 13, 16, 17, and 21), anxiety (items 2, 4, 7, 9, 15, 19, and 20), and stress (items 1, 6, 8, 11, 12, 14, and 18). The DASS-21 has been validated as a reliable and valid tool to assess mental health condition for both clinical and research purposes [14,31,32,33].

## 3. Results

### 3.1. Data Analysis Approach

Data analysis was performed in IBM SPSS Statistics for Windows, Version 23 (IBM Corp, Armonk, NY, USA). For the purpose of clarity and conciseness, we recoded some predictive variables by merging certain close category levels with too few participants (e.g., all the other provinces than “Ontario” were grouped into the “other” category for the “region of residence” variable) to strengthen the meaningfulness of our statistical analysis or interpretation. The Pearson inter-item correlation analysis showed that the three items on health condition (i.e., physical health status, mental health status, and sleep quality) were positively correlated with each other (*r*s = 0.54–0.70, *p* < 0.001). The Principal Component Factor analysis extracted a single-factor component (*λ* = 2.24, accounting for 74.80% variance, loading = 0.83–0.90) from these three items. For the purpose of clarity and conciseness, a general health status index was calculated as a composite average score of these three items in the final data analysis. The final data analysis took the following steps: first, we performed univariate analysis of variance models (ANOVAs) to examine the differences in the DASS-21 outcome scores stratified by sociodemographic (Table 1) and COVID-19-related predictive variables (Table 3). Bonferroni correction was applied for all follow-up multiple comparisons, if needed. Second, as per convention [34], variables with a *p* ≤ 0.20 from the ANOVAs were entered into the corresponding multiple linear regression models for depression, anxiety, and stress (see Table 2 and Table 4). Separate regression models were run for sociodemographic and COVID-19-related predictive variables, to ensure statistical power and considering our interest in both sets of variables.

### 3.2. Mental Health Status

The mental health status of the respondents was characterized with the level of severity in the three subscales of the DASS-21. For the depression subscale, 374 (79.40%), 44 (9.34%), and 53 (11.30%) respondents reported normal, mild, and moderate to extremely severe levels of depression, respectively. Similarly, 395 (83.86%), 25 (5.31%), and 51 (10.83%) respondents reported normal, mild, and moderate to extremely severe levels of anxiety, respectively. Finally, 424 (90.02%), 22 (4.67%), and 24 (5.10%) respondents reported normal, mild, and moderate to extremely severe levels of stress, respectively.

### 3.3. Sociodemographic Predictors for Depression, Anxiety, and Stress

Group differences based on sociodemographic variables in mental health outcome variables were analyzed using the univariate ANOVAs (Table 1). The results showed that, across all three outcome variables, health status and perceived discrimination showed significant group differences in depression, anxiety, and stress (*F*s ≥ 5.15, *p*s ≤ 0.006). Specifically, those in poor health status showed higher levels of depression, anxiety, and stress than their counterparts. Post-hoc multiple comparisons on perceived discrimination showed that those who perceived discrimination also reported higher levels of depression, anxiety, and stress than those who were neutral or who disagreed (*p*s ≤ 0.024). In addition, age groups differed in depression and stress (*F*s ≥ 4.10, *p*s ≤ 0.017). Post-hoc multiple comparisons revealed that middle-aged people showed higher level of stress than their counterparts (35–64 vs. ≥ 65, *p* = 0.023). No significant age group difference was found in depression in the post hoc multiple comparison.

Three multiple linear regression models were conducted for depression, anxiety, and stress, respectively, with sociodemographic variables with *p* ≤ 0.20 from the univariate ANOVAs entered as predictive variables. The models explained a significant amount of variance in depression (*R^2^* = 0.31), anxiety (*R^2^* = 0.26), and stress (*R^2^* = 0.31), *F*s ≥ 22.99, *p*s < 0.001. Across the three models, both health status and perceived discrimination were identified as significant predictors (absolute value of *β*s = 1.19–7.11, *p*s < 0.05). Specifically, those in poor health or who perceived anti-Chinese discrimination reported higher levels of depression, anxiety, and stress. Income level also negatively predicted depression (absolute value of *β*s = 1.65–1.70, *p*s < 0.05). Those with lower incomes tended to score higher in depression relative to those with middle or high incomes. In addition, people in the middle-aged group reported higher levels of stress than those in the younger group (*β =* 1.65, *p* = 0.049).

### 3.4. COVID-19-Related Predictors for Depression, Anxiety, and Stress

The results of univariate ANOVAs on the mental health outcomes stratified by the COVID-19-related variables (Table 3) showed significant differences based on self-infection worry and information confusion in depression, anxiety, and stress (*F*s ≥ 3.26, *p*s ≤ 0.040). Based on the post hoc multiple comparisons, those who were worried about self-infection of COVID-19 reported higher levels of depression, anxiety, and stress than those who reported neutral or not worried (*p*s < 0.001). Similarly, those who reported having concerns or confusion about the authenticity of the information related to COVID-19 “all the time/often” reported higher levels of depression, anxiety, and stress then those who reported “sometimes” or “occasionally/never” (*p*s ≤ 0.002). Additionally, those who endorsed food/goods stocking also showed higher stress levels than those who did not, *F* = 2.89, *p* = 0.049.

Three multiple linear regression models were conducted on depression, anxiety, and stress respectively, with COVID-19-related variables with *p* ≤ 0.20 from the univariate ANOVAs as predictive variables. The models explained a significant amount of variance in depression (*R^2^* = 0.13), anxiety (*R^2^* = 0.14), and stress (*R^2^* = 0.15), *F*s ≥ 6.24, *p*s < 0.001. Across the three models, self-infection worry and information confusion were identified as significant predictors (absolute value of *β*s = 1.84–3.92, *p*s < 0.05). Specifically, those who were worried about themselves being infected with COVID-19 (worried) or having more frequent concerns or confusion about the COVID-19 information (all the time/often) reported differentially higher levels of depression, anxiety, and stress. In addition, attitudes towards Canadian measures, room cleaning/sanitizing, and food/goods stocking were also predictive of one or more mental health outcomes (absolute value of *β*s = 1.33–1.82, *p*s < 0.05). Specifically, those who were optimistic about the Canadian measures reported lower levels of depression relative to those who were neutral about Canadian measures. Individuals who endorsed “room cleaning/sanitizing” behaviour also showed higher levels of anxiety, and those who endorsed “food/goods stocking” behaviour also showed higher levels of stress, compared to their corresponding counterpart groups.

## 4. Discussion

The current study sought to examine the mental health condition of Mainland Chinese in Canada during the peak of the first wave of the COVID-19 pandemic. It also aimed to identify the associated sociodemographic and COVID-19-related predictors. Overall, 11.30%, 10.83%, and 5.10% of respondents reported moderate-to-severe levels of depression, anxiety, and stress respectively. Several sociodemographic (i.e., age, income level, health status, and perceived discrimination) and COVID-19-related predictors (self-infection worry, information confusion, attitude towards Canadian measures, food/goods stocking, or room cleaning/sanitizing behaviour endorsement) were identified for one or more mental health outcomes of the target population. 

In the current study, a majority of respondents reported normal levels of depression, anxiety, and stress. The proportions of those who reported moderate-to-severe depression and anxiety levels (11.30% for depression and 10.83% for anxiety) were slightly lower compared to the data reported by individuals in Mainland China (16.50% for depression and 28.80% for anxiety) at the initial stage of the pandemic, as shown in Wang et al. [14]. The differences may be explained by several factors. First, China was more affected by COVID-19 at the early stage of the pandemic. For example, the numbers of confirmed COVID-19 cases and deaths in China far exceeded Canada at the same stage of the pandemic during the first wave [35]. It has been found that people from countries and areas that had been particularly affected by COVID-19 were more likely to experience psychological distress than those from less-affected places [25]. The strikingly rapid increase of the COVID-19 cases may be a threatening factor for people’s mental health. Second, in response to the COVID-19 outbreak, China implemented strict lockdown measures quickly and largely unexpectedly, such as allowing only one person from each household to go outside every two days, and using health codes to track people’s health status and outdoor activity [36]. It has been shown that the quarantine measures taken in China during the first wave of the pandemic were associated with an increased risk of experiencing mental health problems [37]. Therefore, people living in an environment with more stringent lockdown measures may experience greater psychological distress [25]. Third, Wang et al. [14] collected data at the starting point of the lockdown procedure in Wuhan, whereas the data of the current study were collected from April to June in 2020, around 1–2 months following the implementation of the lockdown in Canada. It is also possible that the initial anxiety and shock associated with the COVID-19 outbreak might be lessened over time.

It was found that people with pre-existing mental and physical health conditions showed higher depression and anxiety than healthy individuals, and their conditions may grow worse due to decreased access to physical and mental health care during the pandemic [22,38,39]. Similar to Wang et al. [14], we found that health status was a significant predictor for mental health condition, suggesting that the psychological effects of COVID-19 may be exacerbated by pre-existing health conditions, especially for Chinese immigrants who have reported social or cultural barriers to accessing health care services [8,9,10,40,41]. Income level was a significant predictor of depression, which is in line with the commonly reported link between economic disadvantage and poor mental health [42,43]. The association between income level and mental health may be further strengthened by the increased unemployment rate during the COVID-19 pandemic in Canada [44], where visible minority groups may be particularly influenced as they were more likely to work in industries that were most affected by the pandemic [45]. While previous studies found that younger adults showed the highest level of psychological distress related to COVID-19 [5,20], we found middle-aged people were more stressed than younger adults. This is probably because middle-aged Chinese in Canada typically provide fundamental support (financial or social) for families and communities [46] and are thus most likely to experience exacerbated family/work-related stress during the pandemic. In contrast, most young adults (international students or young immigrants) were largely dependent on their parents for financial support, and less likely to have caregiving responsibilities.

Consistent with the previous finding, that increased perceived discrimination against Chinese was detrimental to the mental health condition of Chinese residents during the pandemic [47,48,49,50], our results showed the concern about increased discrimination against Chinese as a result of the pandemic was associated with higher levels of depression, anxiety, and stress. This urges the government agencies to take further steps to minimize discrimination against visible minority groups during the pandemic.

Adding to Wang et al. [14], we found that self-infection worry and information confusion significantly predicted worse mental health condition across the board. Worry related to self-awareness of/reflection on infection risk may be particularly predictive of mental health condition because the worry may lead to strict restrictions of social activities and gatherings, which may negatively affect mental health. Furthermore, confusion about the authenticity of COVID-19-related information significantly predicted higher levels of depression, anxiety, and stress. This highlights the importance of increasing the information authenticity in media reports about the pandemic to protect or promote mental health condition. In addition, engaging in certain precautionary behaviours also predicts mental health condition. For example, food/goods stocking behaviour endorsement predicts higher stress, whereas room cleaning/sanitizing behaviour endorsement predicts higher anxiety. These findings are consistent with previous studies showing that greater engagement in safety measures was associated with greater psychological distress during the COVID-19 pandemic [21]. However, there are a few studies that found the opposite, where more engagement in precautionary behaviours (e.g., washing hands frequently, wearing a face mask, etc.) was associated with better mental health conditions in Mainland China [14,51]. This might be related to cultural differences, where Chinese collective culture might strongly encourage a willingness to comply with public health measure compliance (and thus promote their mental health). Alternatively, the discrepancy may be related to the differences in the strictness of the public health measures in response to the pandemic between Canada and China. The generally less-strict and slower-paced public health actions in response to the pandemic may bring uncertainties and doubt among Chinese residents in Canada about the effectiveness and efficiency of Canadian public health measures, and they may thus endorse more precautionary behaviours. Under this circumstance, engagement in precautionary behaviours may be an indicator of the degree of uncertainties or worries individuals had about the COVID-19 pandemic in Canada, which was associated with poor mental health condition. In support of this, it was found that optimistic attitudes towards Canadian measures buffer depression.

This study also has a few limitations. First, the current study mainly used a self-report survey to measure psychiatric symptoms without clinical diagnosis. The gold standard for establishing psychiatric diagnosis involves structured clinical interviews and functional neuroimaging [52,53,54], which does not apply to the current study. Second, all the respondents were recruited through a convenient sampling approach, and a majority of the respondents were female, middle-aged, married, well-educated, and living in Ontario. Therefore, our results may not be generalizable to the general Chinese immigrant population in Canada. Third, the current data only reflected mental health condition during the early stage of the pandemic. Future studies may follow up and examine mental health condition in the following waves to track the mental health trajectory across the timelines of the pandemic.

## 5. Conclusions

Overall, the current study provides important insights into our understanding of the mental health condition of the Mainland Chinese in Canada during the early stage of the COVID-19 pandemic. By identifying sociodemographic and COVID-19-related predictors of mental health condition, the results shed important light on the development of more targeted and effective preventions or interventions to mitigate the mental health impacts of the pandemic on minority groups such as Chinese immigrants. The current study also highlighted the need for culturally appropriate psychological prevention and intervention programs to treat or mitigate mental health issues in Chinese communities in Canada during or post the pandemic era.

## Figures and Tables

**Table 3:** COVID-19-related variables and differences in mental health outcomes (depression, anxiety, and stress), *N* = 433.

Variables		*N* (%)	Depression			Anxiety			Stress		
			M (SD)	F	*p*	M (SD)	F	*p*	M (SD)	F	*p*
**Contact history**											
Exposure	Yes	9 (2)	7.17 (5.36)	0.53	0.591	4.17 (3.46)	0.80	0.451	7.50 (5.13)	0.83	0.437
	No	357 (82)	4.60 (5.86)			3.38 (5.03)			5.43 (6.30)		
	Unsure	97 (16)	6.52 (9.12)			4.78 (6.90)			7.73 (9.09)		
Symptoms	Yes	20 (5)	7.71 (7.86)	2.76	**0.098**	5.71 (8.52)	2.42	**0.121**	7.62 (8.33)	0.67	0.413
	No	413 (95)	4.97 (6.64)			3.62 (5.29)			5.90 (6.95)		
Other-contraction	Yes/Suspect	40 (9)	5.72 (8.25)	0.83	0.438	4.88 (7.29)	2.54	**0.080**	7.12 (7.69)	2.92	**0.055**
	No	318 (73)	4.71 (6.29)			3.24 (4.90)			5.38 (6.59)		
	Unsure	75 (18)	6.40 (7.49)			5.11 (6.43)			8.03 (8.04)		
**Risk perception**											
Self-infection prediction	Likely	130 (30)	6.14 (7.29)	0.76	0.467	4.60 (6.65)	0.95	0.388	6.93 (7.35)	1.14	0.321
	Neutral	218 (50)	5.31 (7.08)			3.78 (5.31)			6.31 (7.22)		
	Unlikely	85 (20)	3.28 (4.46)			2.41 (3.56)			4.10 (5.74)		
Self-infection worry	Worried	248 (57)	6.61 (7.65)	3.26	**0.040**	4.93 (6.39)	3.46	**0.032**	7.78 (7.86)	6.23	**0.002**
	Neutral	99 (23)	3.72 (5.09)			2.55 (3.93)			4.13 (5.09)		
	Not worried	86 (20)	2.36 (3.65)			1.57 (2.33)			3.00 (4.33)		
Family-infection worry	Likely	305 (70)	5.90 (7.16)	0.53	0.589	4.37 (6.02)	0.47	0.628	6.91 (7.40)	0.61	0.544
	Neutral	64 (15)	3.66 (5.81)			2.69 (4.09)			4.42 (6.18)		
	Unlikely	64 (15)	2.67 (4.04)			1.59 (2.40)			3.08 (4.45)		
Attitude towards Canadian measures	Optimistic	147 (34)	3.48 (5.12)	1.74	**0.177**	2.56 (3.99)	1.50	0.223	4.44 (5.49)	1.61	0.202
	Neutral	206 (38)	5.92 (7.41)			4.41 (6.23)			6.91 (7.78)		
	Pessimistic	80 (18)	6.20 (3.08)			4.22 (5.62)			6.70 (7.16)		
Threat perception	Agree	359 (83)	5.47 (7.07)	0.63	0.534	4.08 (5.82)	0.13	0.874	6.52 (7.34)	0.594	0.553
	Neutral	57 (13)	3.09 (4.15)			2.11 (2.96)			3.43 (4.40)		
	Disagree	17 (4)	4.00 (4.64)			1.41 (1.97)			3.29 (4.52)		
Information confusion	All the time/Often	115 (27)	7.66 (8.64)	6.15	**0.002**	5.55 (7.33)	5.12	**0.006**	8.43 (8.94)	4.41	**0.013**
	Sometimes	211 (49)	4.55 (5.93)			3.33 (4.68)			5.61 (6.27)		
	Occasional/Never	107 (24)	3.59 (5.10)			2.62 (4.16)			4.29 (5.41)		
**Perceived measure effectiveness**											
Mask wearing	Completely effective	178 (41)	5.87 (7.62)	0.84	0.433	3.54 (6.64)	0.84	0.435	6.88 (7.77)	0.35	0.703
	Somewhat effective	223 (52)	4.55 (5.95)			3.22 (4.32)			5.47 (6.46)		
	Average or ineffective	32 (7)	4.22 (5.63)			2.23 (4.29)			4.28 (5.39)		
Taking vitamin C or other supplements	Effective	155 (36)	5.28 (6.98)	0.92	0.398	4.25 (6.19)	1.72	**0.179**	6.21 (7.27)	0.70	0.497
	Average	206 (48)	5.33 (7.07)			3.79 (5.34)			6.00 (7.23)		
	Ineffective	72 (16)	4.39 (5.11)			2.37 (3.84)			5.89 (6.00)		
Gargling with salt water	Effective	169 (39)	5.46 (7.30)	0.66	0.518	4.49 (6.67)	2.61	**0.075**	6.37 (7.70)	1.55	0.214
	Average	190 (44)	4.98 (6.50)			3.18 (4.65)			5.55 (6.75)		
	Ineffective	74 (17)	4.76 (6.19)			3.22 (4.18)			6.24 (6.18)		
**Precautionary measure endorsement**											
Food/Goods stocking	Yes	312 (72)	5.67 (7.06)	1.50	0.221	4.23 (6.01)	1.32	0.252	6.75 (7.33)	3.89	**0.049**
	No	121 (28)	3.71 (5.11)			2.42 (3.43)			4.14 (5.26)		
Room cleaning /sanitizing	Yes	307 (71)	5.67 (7.18)	0.75	0.386	4.33 (6.13)	2.00	**0.158**	6.80 (7.60)	2.05	**0.153**
	No	126 (29)	3.88 (5.35)			2.29 (3.09)			4.18 (4.95)		

Note. Bold *p* values (*p* ≤ 0.20) refer to the variables to be entered in the regression models displayed in Table 4.

**Table 4:** Linear regression models for mental health outcomes (i.e., depression, anxiety, and stress).

Predictors		Depression (*N* = 471)		Anxiety (*N* = 462)		Stress (*N* = 454)	
		β	95% CI	β	95% CI	β	95% CI
**Contact History**							
Symptoms	Yes (reference)						
	No	−2.31	−5.11, 0.50	−1.90	−4.26, 0.47	X	X
Other-contraction	Yes/Suspect (reference)						
	No	X	X	−1.12	−2.79, 0.56	−0.97	−3.06, 1.12
	Unsure	X	X	0.64	−1.32, 2.59	1.64	−0.80, 4.07
**Risk Perception**							
Self-infection worry	Worried (reference)						
	Neutral	−2.13 **	−3.58, −0.68	−1.95 ***	−3.15, −0.75	−3.11 ***	−4.59, −1.62
	Not worried	−3.45 ***	−4.99, −1.91	−2.62 ***	−3.92, −1.32	−3.68 ***	−5.30, −2.06
Attitude towards Canadian measures	Optimistic (reference)						
	Neutral	1.82 *	0.52, 3.13	X	X	X	X
	Pessimistic	1.44	−0.25, 3.14	X	X	X	X
Information confusion	All the time/Often (reference)						
	Sometimes	−2.67 ***	−4.10, −1.24	−1.84 **	−3.00, −0.68	−1.92 *	−3.38, −0.47
	Occasional/Never	−2.97 ***	−4.61, −1.33	−2.09 **	−3.44, −0.74	−2.69 **	−4.38, −1.01
**Perceived measure effectiveness**							
Taking vitamin C or other supplements	Effective (reference)						
	Average	X	X	0.48	−0.85, 1.82	X	X
	Ineffective	X	X	−1.13	−3.02, 0.77	X	X
Gargling with salt water	Effective (reference)						
	Average	X	X	−1.02	−2.36, 0.33	X	X
	Ineffective	X	X	−0.03	−1.93, 1.87	X	X
**Precautionary measure endorsement**							
Food/Goods stock	Yes (reference)						
	No	X	X	X	X	−1.70 *	−3.11, −0.28
Room cleaning /sanitizing	Yes (reference)						
	No	X	X	−1.33 *	−2.43, −0.22	−1.25	−2.67, 0.17

Note: Listwise method was used to handle missing values. CI = Confidence of Interval. “X” cells refer to the variables not included in the regression model based on the ANOVA analysis in Table 3. * *p* < 0.05, ** *p* < 0.01, *** *p* < 0.001.

## Data Availability

The data files, the SPSS syntax file, and the variable label index file can be retrieved from https://doi.org/10.17605/OSF.IO/9KE7V (accessed on 6 November 2021).
